# The ectomycorrhizal fungus *Scleroderma bovista* improves growth of hazelnut seedlings and plays a role in auxin signaling and transport

**DOI:** 10.3389/fmicb.2024.1431120

**Published:** 2024-08-07

**Authors:** Yunqing Cheng, Siyu Sun, Hanxiao Lou, Yutong Dong, Hongli He, Qi Mei, Jianfeng Liu

**Affiliations:** Jilin Provincial Key Laboratory of Plant Resource Science and Green Production, Jilin Normal University, Siping, China

**Keywords:** hazel, ectomycorrhizal fungus, comparative transcriptome, *Scleroderma bovista*, growth

## Abstract

**Introduction:**

*Scleroderma bovista* can form symbiotic ectomycorrhizal fungi with hazel roots. The mechanism through which *S. bovista* promotes hazelnut growth remains unclear.

**Methods:**

This study aimed to evaluate the effect of ectomycorrhizal fungus *S. bovista* on the growth and development of hazel roots and gene expression changes through comparative transcriptome analysis.

**Results:**

After inoculation with *S. bovista*, the fungus symbiotically formed ectomycorrhiza with hazel roots. The fresh weights of the aboveground and underground parts of My treatment (inoculated with S. bovista and formed mycorrhiza) were much higher than those of the control, respectively. The length, project area, surface area, volume, forks, and diameter of the inoculated seedlings root were 1.13 to 2.48 times higher than those of the control. In the paired comparison, 3,265 upregulated and 1,916 downregulated genes were identified. The most significantly enriched Gene Ontology term for the upregulated Differentially Expressed Genes was GO:0005215 (transporter activity). Immunohistochemical analysis suggested that the expression levels of auxin and Auxin Response Factor9 were significantly increased by *S. bovista* after the formation of mycorrhizal fungi in hazelnut root tips.

**Discussion:**

These results indicate that genes related to auxin biosynthesis, transport and signaling, and transport of nutrients may contribute to root development regulation in hazel ectomycorrhiza.

## 1 Introduction

Mycorrhizal fungi are highly evolved mutualistic symbionts formed between soil fungi and plant roots. In this mutually beneficial symbiosis, the host plant absorbs mineral nutrients from outside the root system through symbiotic fungal hyphae, and the fungi assimilate photosynthetic carbon through the host plant (Harley, 1988; Cheng et al., 2021; Zou et al., 2021). Approximately 5 billion tons of photosynthetic products are fixed in the soil yearly through mycorrhizal fungi worldwide, making it significant in the carbon and nitrogen balance of the entire ecosystem (Jiang et al., 2017). The role of mycorrhizal symbionts in improving plant uptake and accumulation of soil mineral nutrients, promoting plant resistance to drought, waterlogging, salt, disease, and heavy metal stress in ecosystems has been recognized. The families and genera of important woody plants with ectomycorrhizal fungi include Betulaceae, Rhamnaceae, Rosaceae, Casuarinaceae, and Dipterocarpaceae (Corrales et al., 2022). Ectomycorrhizal fungi have been found in plants such as *Alnus*, *Betula*, *Carpinus*, *Ostrya*, *Ostryopsis*, and *Corylus* in the Betulaceae. Hazelnut is a species of the *Corylus* genus in the family Betulaceae and is one of the world’s four major nuts. The hazelnut industry is significant in supporting the economic development of Northeast China, Shandong and Hebei mountainous areas (Liu et al., 2014). Although the existence of ectomycorrhizal fungi in hazelnuts has been confirmed, studies on hazelnut mycorrhizal fungi in China are lacking, and there is no corresponding microbial agent for production practices, which is not conductive to hazel industrial development.

Hazelnut mycorrhizal fungi in Northeast China were recently discovered (Cheng et al., 2023). Though inoculation of hazel mycorrhiza samples and repeatedly colonies culture, a pure colony of *Scleroderma bovista* was obtained. Microbial diversity analysis of the rhizosphere soil, paraffin sectioning, sequencing, and sequence comparison showed that *S. bovista* can form symbiotic ectomycorrhizal fungi with hazel roots. Outdoor potted cultivation of hazel seedlings and inoculation with *S. bovista* and were performed, and it was found that *S. bovista* can strongly promote the growth of the aboveground and underground parts of hazel seedlings (Cheng et al., 2023). Based on these results, it is assumed that the natural distribution of *S. bovista* and hazel production areas in China overlap. It is speculated that *S. bovista* can form a stable symbiotic relationship with the hazel root system in production areas and may be vital in promoting root development, plant growth, and improving stress tolerance. *S. bovista* is an edible medicinal fungus with hemostatic and detoxifying effects and a high medicinal value. Its tender fruiting body is a delicious mushroom. *S. bovista* needs to form symbiotic relationships with trees to form fruiting bodies (Zhang et al., 2021). Therefore, establishing a symbiotic relationship between *S. bovista* and hazel trees benefits the growth and development of hazel trees, the development of a hazel understory economy, and improves cultivation. Thus, *S. bovista* is a potentially essential ectomycorrhizal fungus urgently required for developing the hazel industry.

The formation of ectomycorrhiza is a common ecological phenomenon in nature. Ectomycorrhizal fungi hyphae can extend into the root cortex cells to form a fungal mesh, while spreading on the root surface to form a hyphal sheath, replacing the role of root hairs and absorbing nutrients and water. This structure not only improves the water and nutrient metabolism of plants, but also increases the absorption of trace elements such as copper and zinc by the host plant roots, decomposes organic matter in the soil, accelerates soil nutrient cycling, improves soil physicochemical properties, and enhances the effectiveness of nutrients in the soil (Kuyper and Suz, 2023). Ectomycorrhizal fungi can also promote plant growth by enhancing the absorption of nutrients by trees, which is one of the important mechanisms for promoting plant growth. Especially in low soil phosphorus concentrations, ectomycorrhizal fungi enable plants to more effectively absorb phosphorus from the soil, thereby promoting plant growth (Almeida et al., 2023). However, the mechanism through which *S. bovista* promotes hazelnut growth remains unclear. Therefore, this study aimed to evaluate the effects of the ectomycorrhizal fungus *S. bovista* on the growth and development of hazelnuts and gene expression changes through comparative transcriptome analysis.

## 2 Materials and methods

### 2.1 *Scleroderma bovista* inoculation and measurement of root growth indicators

In 2023, seeds of the hazel (*Corylus heterophylla* × *Corulys avellana*) cultivar “Yuzhui” and our self-isolated and purified strain of *S. bovista* were chosen for this study (Cheng et al., 2023). Hazelnut seeds were first stratified in a 4°C refrigerator for 2 months and soaked overnight with 500 mg/L GA_3_ (Gibberellin A3) to further break dormancy. Seed accelerating germination was performed at 25°C in an illuminating incubator for 2 weeks, and 50 seeds with a root length of 3–5 cm were used for sowing in early May. Hazel seeds were sown in plastic pots containing 6 kg of sterilized humus soil, with 25 pots of the control (inoculated with autoclaved *S. bovista*) and 25 of My treatments (inoculated with *S. bovista* and formed mycorrhiza) in May. The sowing depth was 5 cm. In My treatment, *S. bovista* was cultured in dishes containing MMN medium (Modified Melin-Norkrans Medium) according to the description of a previous reference, and 10 *S. bovista* cubes with a side length of 1.0 cm were inoculated into each pot (Cheng et al., 2023). Potted seedlings were cultivated outdoors at Jilin Normal University (43°9′18″N, 124°20′11″E). Among the 25 treated seedlings, eight were used to measure growth indicators, and six to extract root RNA. Subsequently, 4 months after sowing, the seedlings were carefully removed from plastic pots in September. After removing the soil adhering to the roots, a WinRhino root analysis system (Regent Instruments Inc., Canada) was used to measure root indicator differences between the control and My treatments. Paraffin sections and mycorrhiza staining were performed according to a previously described method (Cheng et al., 2023).

### 2.2 RNA sequencing

Six seedlings each in the control and My treatments were used to extract RNA in late July. At this moment, the growth-promoting effect of ectomycorrhizal fungi was already evident. The roots of control and My treatments were sampled, and six digital gene expression libraries were constructed to investigate gene expression differences, including Ck-1, Ck-2, Ck-3, My-1, My-2, and My-3. There were two treatments (Ck and *S. bovista* inoculation) in our experiments, three biological replicates in each treatment, and two seedlings in each biological replicate. The RNA EasySpin Isolation System (AiDLab Biotech, Beijing, China) was used to extract total RNA from fresh root samples following the product manual. Post-processing and sequencing of the RNA samples were performed according to our previous reports (Cheng et al., 2023). Sequencing was performed on an Illumina NovaSeq 6000 platform, and raw transcriptome data were deposited in the Sequence Read Archive^1^ under the access number of PRJNA1061797.

### 2.3 Bioinformatic analysis of transcriptome data

Clean data were generated by filtering the raw data, removing adapters, sequences containing non-AGCT bases at the 5′ end, sequences with a quality value less than those of Q20, reads with N content of more than 10%, and small fragments with a length less than 25 bp. HISAT2,^2^ an alignment program for mapping next-generation sequencing reads, was used to align clean data with the hazel reference genome^3^ (Liu et al., 2021; Zhang et al., 2021). The obtained RNA-Seq alignments were assembled into transcripts using StringTie software^4^ (Shumate et al., 2022). The obtained transcripts were aligned to six public databases to acquire annotation information, including the NR, Swiss Prot, Pfam, STRING, gene oncology (GO), and Kyoto Encyclopedia of Genes and Genomes (KEGG) databases. StringTie was used to calculate the fragments per kilobase of transcript per million fragments mapped (FPKM) value of each gene in the sample based on the alignment results of the HISAT2 software, representing the expression level of the gene in the samples, and DESeq2 was used to identify differentially expressed genes (DEGs) (Love et al., 2014; Zhang et al., 2021; Shumate et al., 2022). The criteria for determining DEGs were set as |Log_2_Fold Change| >1.00 and false discovery rate (FDR) <0.05. Fold Change refers to the value obtained by dividing the expression level of sample My by the expression level of sample Ck, and FDR refers to the false discovery rate. Hierarchical clustering analysis of the DEGs was performed using Multi Experiment Viewer.^5^ ClusterProfiler software (3.4.4) was used to perform GO and KEGG pathway enrichment analysis of DEGs, and the criteria for determining significantly enriched GO terms and DEGs KEGG pathway were set as *p*-value <0.05 and adjusted *p*-value <0.05.

### 2.4 qRT-PCR

Twenty DEGs (10 each of upregulated and downregulated) were randomly chosen to perform further qRT-PCR analysis, and the detailed primer sequences used in the analysis are listed in Supplementary Table S1. *Beta actin* was used as a reference gene in the analysis, and the fold changes (FCs) of the chosen genes were calculated using 2^−ΔΔCt^ method (Schmittgen and Livak, 2008).

### 2.5 Immunohistochemical analysis

*S. bovista* cultured *in vitro* in auxin free medium and medium containing 2.0 mg/L auxin, the ectomycorrhiza and non-mycorrhiza root tips of hazelnuts were fixed in a 4% paraformaldehyde solution. Subsequently, the root tip samples were subjected to ethanol gradient dehydration, paraffin embedding, and sectioning, with a slice thickness of 8 μM. The paraffin slices were spread flat at 36°C and dried at 25°C. After confirming the slice integrity through microscopic examination, immunohistochemical analysis was performed. The primary process of immunohistochemical analysis was performed as previously described (Wei et al., 2021). A monoclonal anti-auxin antibody (SIGMA, United States) was used for immunohistochemical analysis of auxin. A specific antibody against a 390 amino acid (aa) long peptide sequence (10–400 aa) containing unique regions in ARF9 (Cor0156840.1) was synthesized by ABclonal Biotechnology Co., Ltd. (Wuhan, China). The secondary antibody in the negative control was distilled water, and that in the experimental group was labeled with Horseradish Peroxidase. The chromogenic agent used for immunohistochemical analysis of the auxin and ARF9 was 3, 3-N-Diaminobenzidine Tertrahydrochloride DAB. Horseradish peroxidase can catalyze DAB to produce brown precipitates, indicating the expression position and levels of auxin and ARF9 in the tissues. Finally, a light microscope (Axio Imager M2; Carl Zeiss, Jena, Germany) was used to observe and record the expression and distribution of auxins and ARF9.

### 2.6 Statistical analysis

Analysis of variance using SAS software version 8.01 (SAS Institute, Inc., Cary, NC, United States) was used to determine significant differences between the control and My treatments. The means of the data acquired from the control and My treatments were compared using the least significant difference test at a 5% significance level.

## 3 Results

### 3.1 Effect of *Scleroderma bovista* inoculation on seedling growth

**Figure 1:**
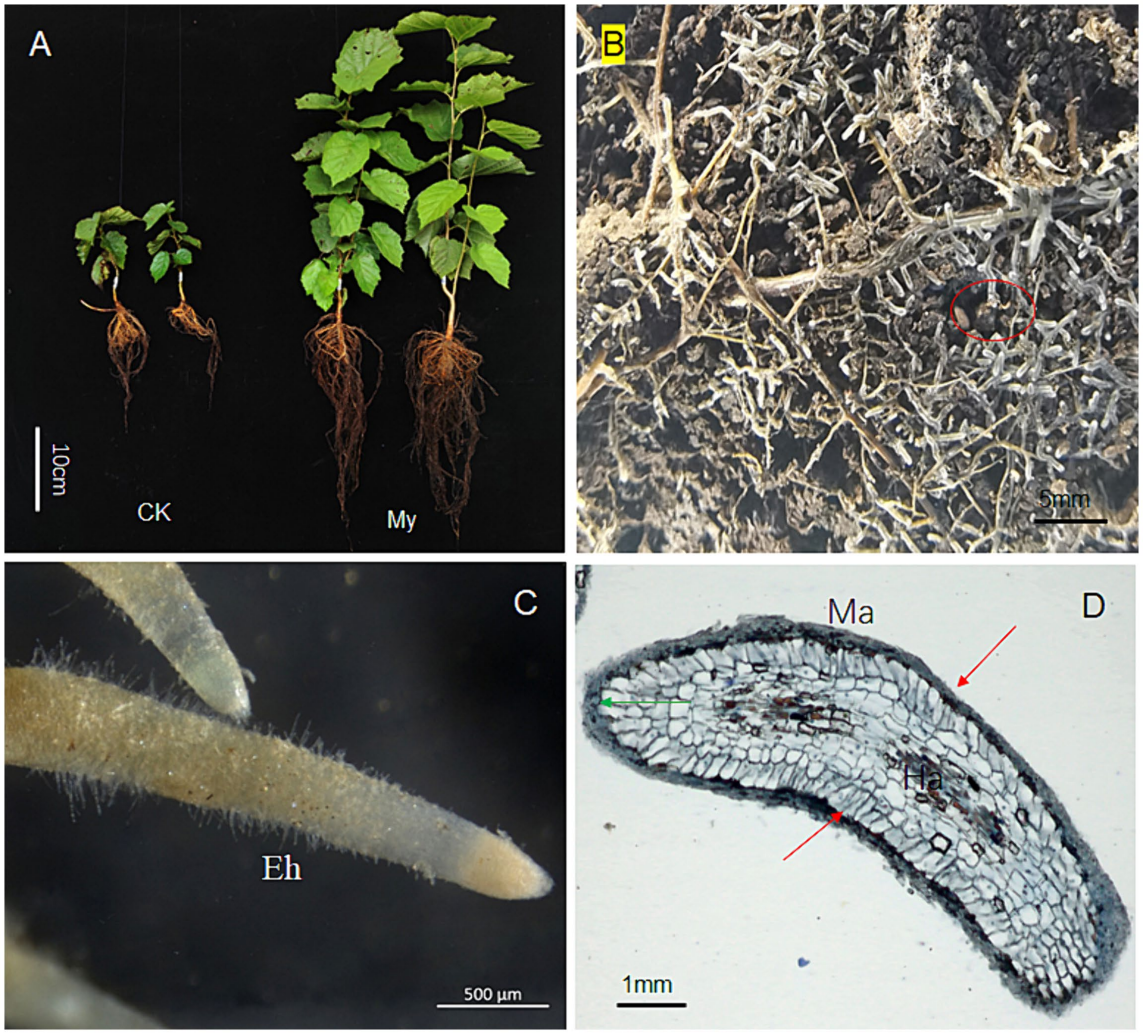
The effect of inoculation with *Scleroderma bovista* on the growth and root development of hazelnut seedlings. **(A)** Inoculated seedlings with *S. bovista* and non-inoculated control. My, seedling with mycorrhiza. Ck, control. **(B)** Mycorrhiza of seedling inoculated with *S. bovista*. This picture was taken from the pot bottom. The red ellipse indicates white mycorrhiza. **(C)** Mycorrhiza of seedling inoculated with *S. bovista*. This picture was taken with a light microscope of Axio Imager M2. **(D)** Longitudinal section of mycorrhizal fungi. The green arrow indicates the root cap, while the red arrow indicates the ectomycorrhizal fungi mantle wrapped outside the root tip. Ha, Hartig net; Ma, mantle; Eh, external hyphae.

*S. bovista* inoculation significantly promotes hazelnut seedling growth (Figure 1). After growing outdoors for 4 months, My treatment (inoculated with *S. bovista* and formed mycorrhiza) seedlings had significantly higher plant height, larger leaf area, and more developed root systems (Figure 1A). In September, many roots showed mycorrhiza formation at the bottom of the potted soil during the My treatment. Ectomycorrhiza is white-colored, and *S. bovista* only coexists with the hazelnut root system at the root tip, forming a white mantle with a length of only 3–5 mm (Figures 1B,C). Paraffin sectioning and staining of mycorrhiza revealed that the mantle was wrapped around the root tip of hazel, with a thickness of approximately 100 μm (Figure 1D). In addition, we observed Hartig nets in the intercellular areas of the cortex (Figure 1D). Mantles and Hartig nets are typical ectomycorrhizal structures.

### 3.2 Effect of *Scleroderma bovista* inoculation on root development

**Figure 2:**
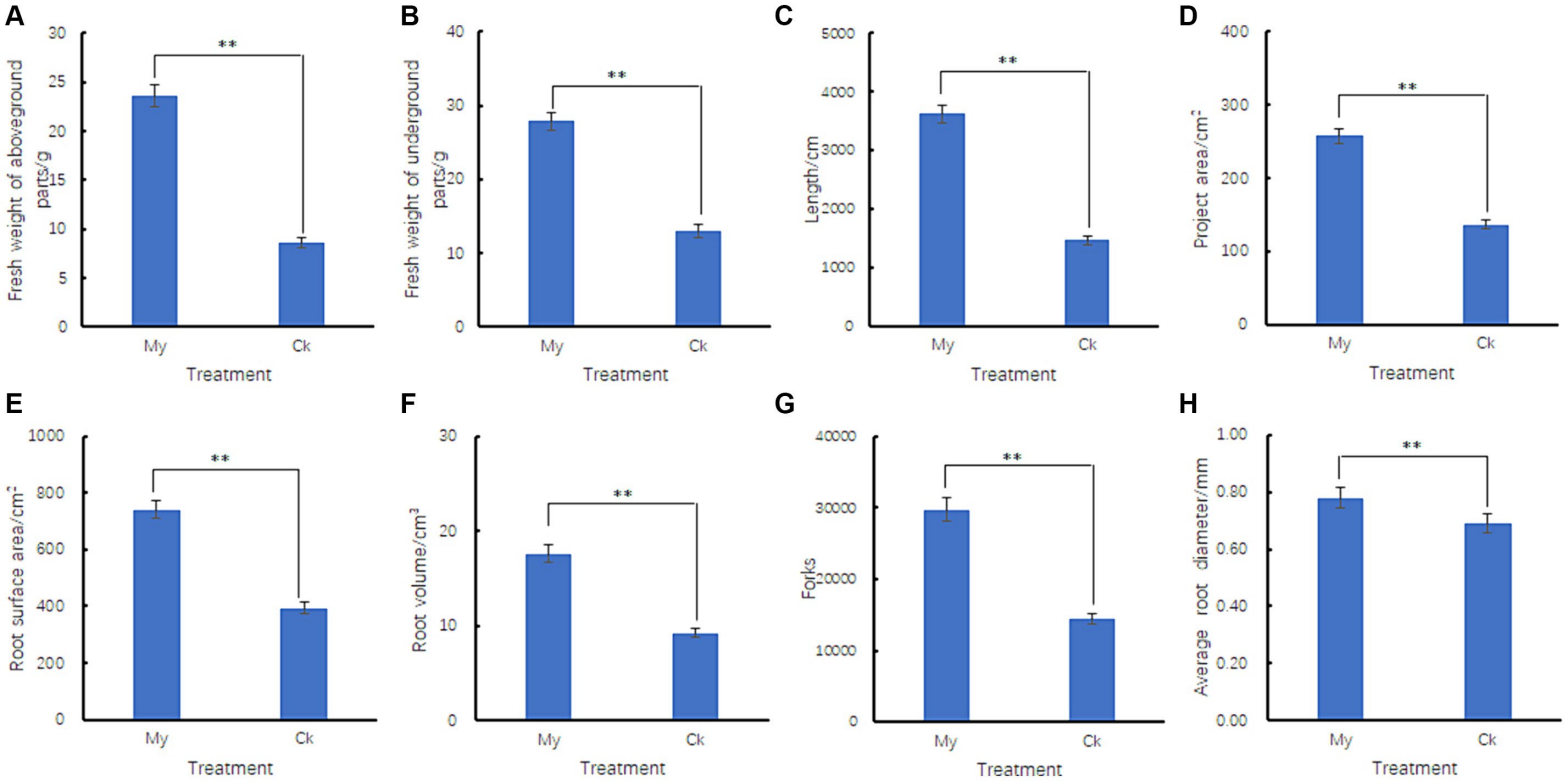
Effect of *Scleroderma bovista* inoculation on the root indexes of hazel. Comparison of fresh mass of aboveground parts **(A)**, underground parts **(B)**, root length **(C)**, root project area **(D)**, root surface area **(E)**, root volume **(F)**, root forks **(G)** and root average diameter **(H)** between control and *S. bovista* inoculation treatment. CK, non-inoculated control. My, treatments inoculated with *S. bovista* and formed mycorrhiza. ^**^Indicates significant difference at *p* ≤ 0.05 as indicated by LSD test.

The fresh weights of the aboveground and underground parts of the inoculated seedlings were 2.73 and 2.15 times higher than those of the control (Figures 2A,B), respectively, and there was a significant difference between the two treatments, indicating that the inoculated seedlings grew faster. The length, project area, surface area, volume, forks, and diameter of the inoculated seedling roots were 2.48, 1.88, 1.88, 1.90, 2.06, and 1.13 times higher than those of the control, respectively (Figures 2C–H), and the root indicators between these two treatments reached a significant level (*p <* 0.05). These results indicate that *S. bovista* inoculation significantly affects root development. After *S. bovista* inoculation, the absorption area of hazel seedlings increased, and the root system branched more. These changes significantly enhanced the water and nutrient absorption capacities of the hazelnut seedlings.

### 3.3 RNA sequencing, sequence mapping, and transcript expression

**Figure 3:**
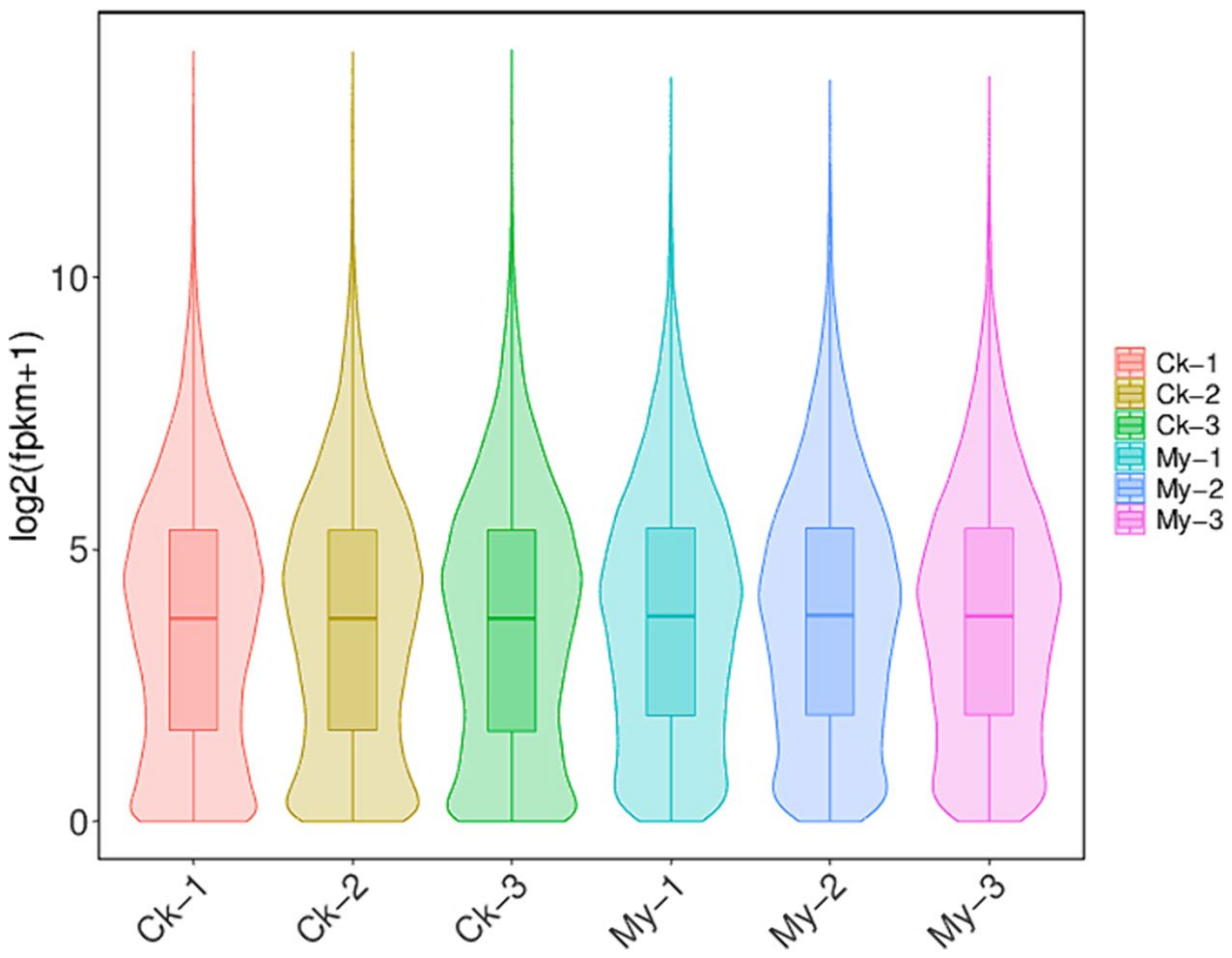
Violin chart of gene expression levels. CK, non-inoculated control. My, treatments inoculated with *S. bovista* and formed mycorrhiza. The numbers after CK and My represent biological replicates.

Six biological samples were sequenced using the Illumina NovaSeq 6000 platform. A total of 712,889,086 raw reads were generated, covering a length of 106,933,362,900 bp. All raw sequencing data were deposited in the sequence read archive under accession number PRJNA1061797. After filtering for low-quality sequences, we obtained 712,369,050 clean reads covering 102,081,099,845 bp. Clean reads were mapped to the hazel reference genomes (Liu et al., 2021), and 564,217,598 reads could be mapped to the reference genome. Among the 166,493,090 clean reads from the three control samples, 149,071,936 were mapped to the reference genome, accounting for 89.53% of all clean reads. Among the 397,724,508 clean reads from the three My treatment samples, 229,725,428 reads were mapped to the reference genome, accounting for 57.75% of all the clean reads. There were 22,319 protein-coding genes in the hazel genome. In the Ck treatments, 18,527 genes were expressed in the roots, with an average FPKM value of 42.26 (Supplementary Table S2). In My treatments, 19,292 genes were expressed in the roots, with an average FPKM value of 44.18 (Supplementary Table S2). Therefore, My treatments resulted in the expression of more genes and higher expression levels than Ck treatments. We drew a violin chart of the expression levels of various samples to explore the overall effect of *S. bovista* inoculation on the FPKM distribution of root genes in hazels. The median FPKM of My treatments was very close to that of Ck, and the lower quartile of My treatments was significantly higher than that of Ck (Figure 3). These results indicate that *S. bovista* inoculation treatment increased the number of expressed genes in hazel roots and significantly increased the expression levels of low-abundance genes.

### 3.4 DEGs

**Figure 4:**
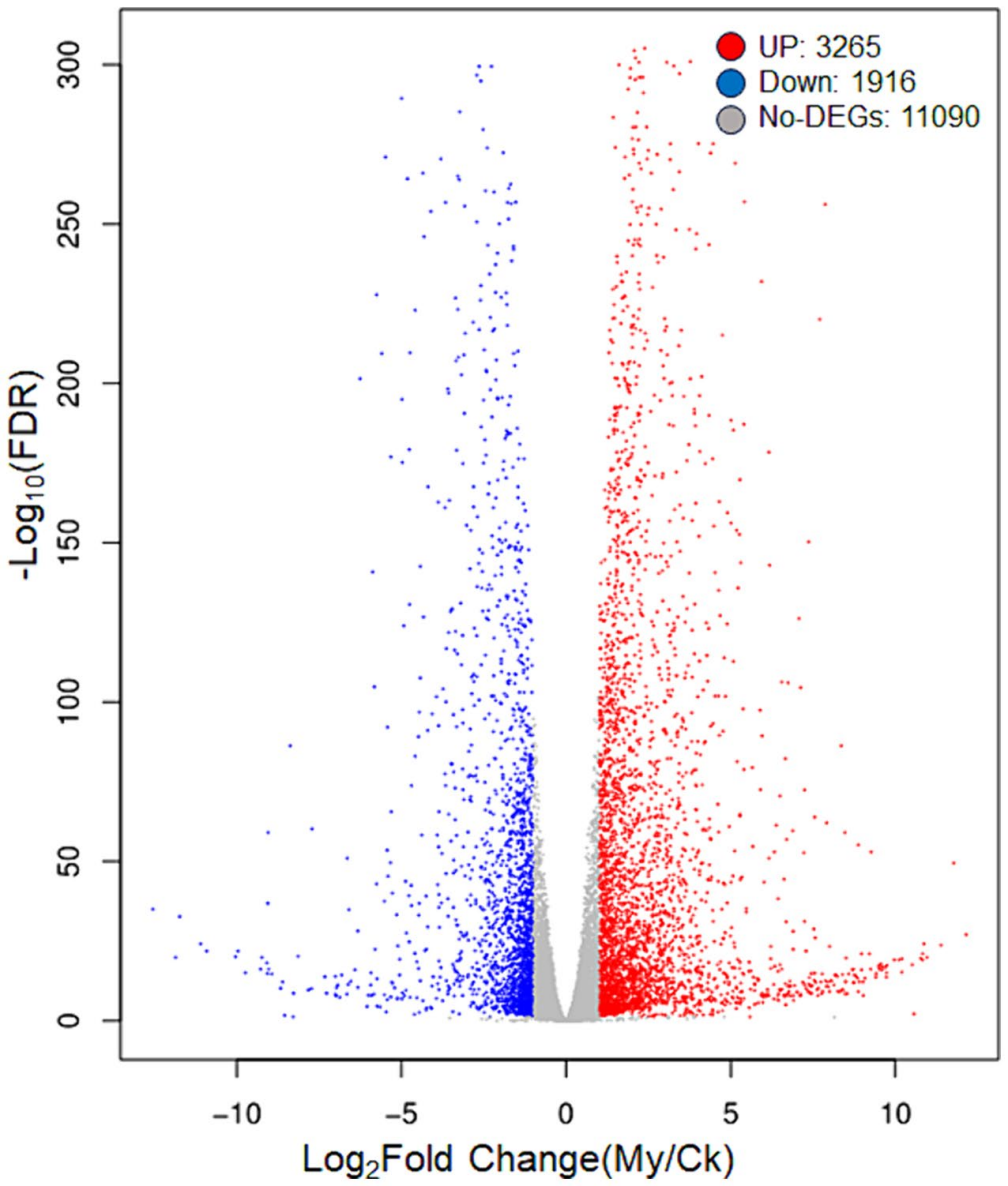
A volcanic map of differentially expressed genes. CK, non-inoculated control. My, treatments inoculated with *S. bovista* and formed mycorrhiza. The horizontal axis represents the gene expression difference between two samples, which is the value obtained by dividing the expression level of sample My by the expression level of sample Ck. The vertical axis represents the *p*-value, and the higher the *p*-value, the more significant the expression difference. The values in the horizontal and vertical axes have been logarithmized. Each dot in the graph represents a specific gene, with red dots indicating significantly upregulated genes, blue dots indicating significantly downregulated genes, and black dots indicating non significantly differentially expressed genes.

The criteria |Log_2_Fold Change| >1.00 and FDR <0.05 were set to determine DEGs in paired comparison of My vs. Ck. Compared with the Ck treatment, 3,265 upregulated and 1,916 downregulated genes were identified in the My treatment (Figure 4). The number of upregulated genes was 1.70 times that of the downregulated, suggesting that *S. bovista* inoculation improves gene expression levels. All DEGs and their corresponding annotations are presented in Supplementary Table S3.

### 3.5 Hierarchical clustering analysis of DEGs

**Figure 5:**
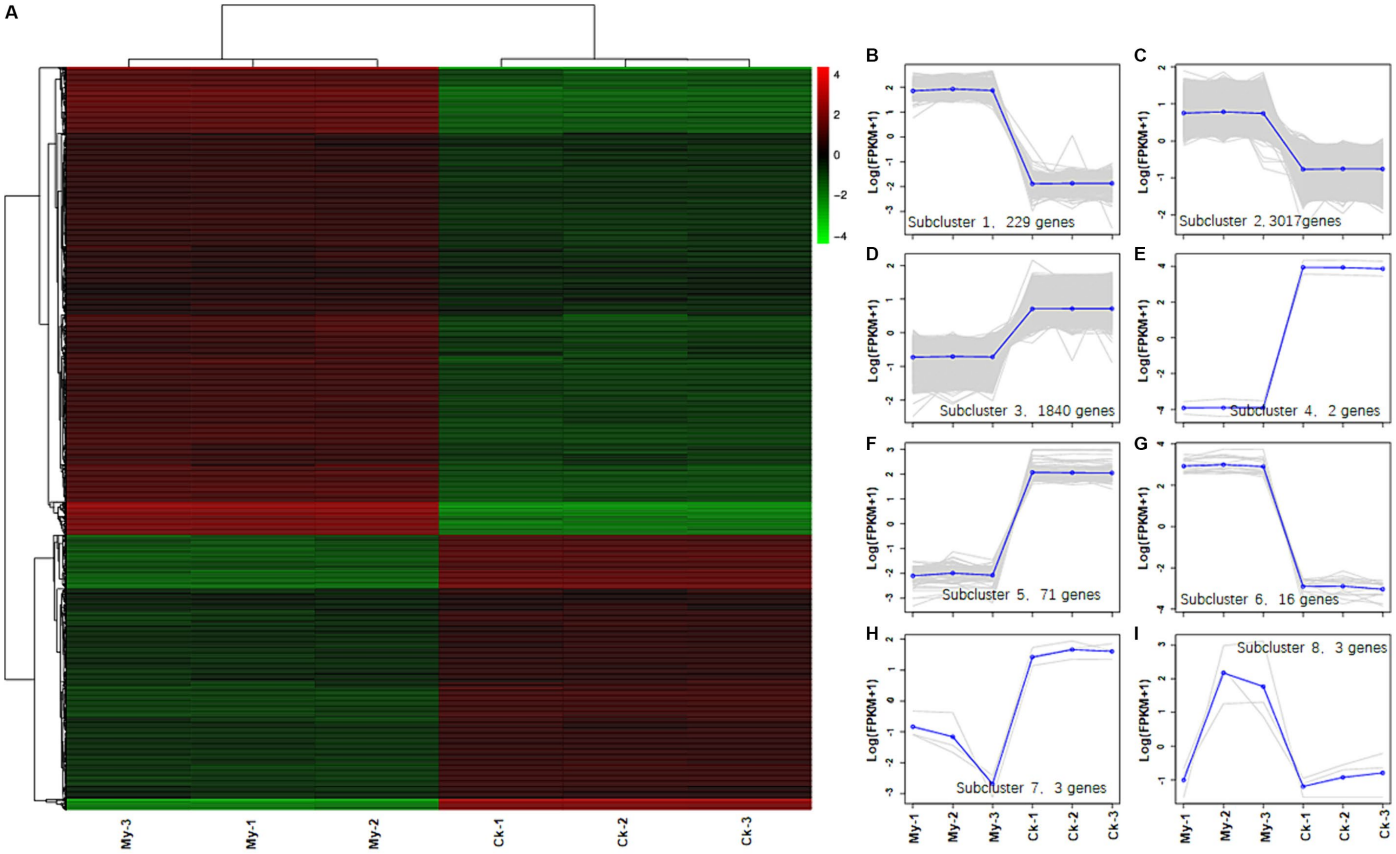
Heatmap analysis of differentially expressed genes between control, *Scleroderma bovista* inoculation treatment and trend analysis of eight subclusters. **(A)** Heatmap analysis of differentially expressed genes between control and *S. bovista* inoculation. Each column in the left graph represents a sample, and each row represents a gene. The color in the graph represents the expression level of the gene in the group of samples (Log_10_FPKM), while red represents a higher expression level of the gene in the sample and green represents a lower expression level. Each line in the right figure represents a gene, and the blue line represents the average expression level of all genes in the subcluster. Each right graph displays a type of expression pattern, which reflects the trend of changes in the expression level of this group of genes. **(B)** Subcluster 1. **(C)** Subcluster 2. **(D)** Subcluster 3. **(E)** Subcluster 4. **(F)** Subcluster 5. **(G)** Subcluster 6. **(H)** Subcluster 7. **(I)** Subcluster 8. CK, non-inoculated control. My, treatments inoculated with *S. bovista* and formed mycorrhiza. The numbers after CK and My represent biological replicates.

Hierarchical clustering analysis of DEGs was used to group genes with the same or similar expression patterns, revealing the function of unknown genes or the unknown functions of known genes. A gene expression heatmap was used to visualize the differences in the expression levels of the DEGs between the control and My samples (Figure 5A). All 5,181 DEGs were divided into eight subclusters (Supplementary Table S4), each containing 229 (Figure 5B, subcluster 1), 3,017 (Figure 5C, subcluster 2), 1,840 (Figure 5D, subcluster 3), 2 (Figure 5E, subcluster 4), 71 (Figure 5F, subcluster 5), 16 (Figure 5G, subcluster 6), 3 (Figure 5H, subcluster 7), and 3 (Figure 5I, subcluster 8) DEGs. Therefore, Sub-cluster 2 contained the most genes, followed by subclusters 3 and 1. The three subclusters accounted for 98.17% of the total DEGs and exhibited similar expression patterns. In subclusters 1 and 2, the gene expression level of the My treatments was higher than that of the control, whereas in subcluster 3, the gene expression level of the My treatments was lower than that of the control. Similarly, we deduced that these differences in gene expression levels might cause the promotion of hazel root growth through *S. bovista* inoculation.

### 3.6 GO enrichment analysis of upregulated and downregulated DEGs

**Figure 6:**
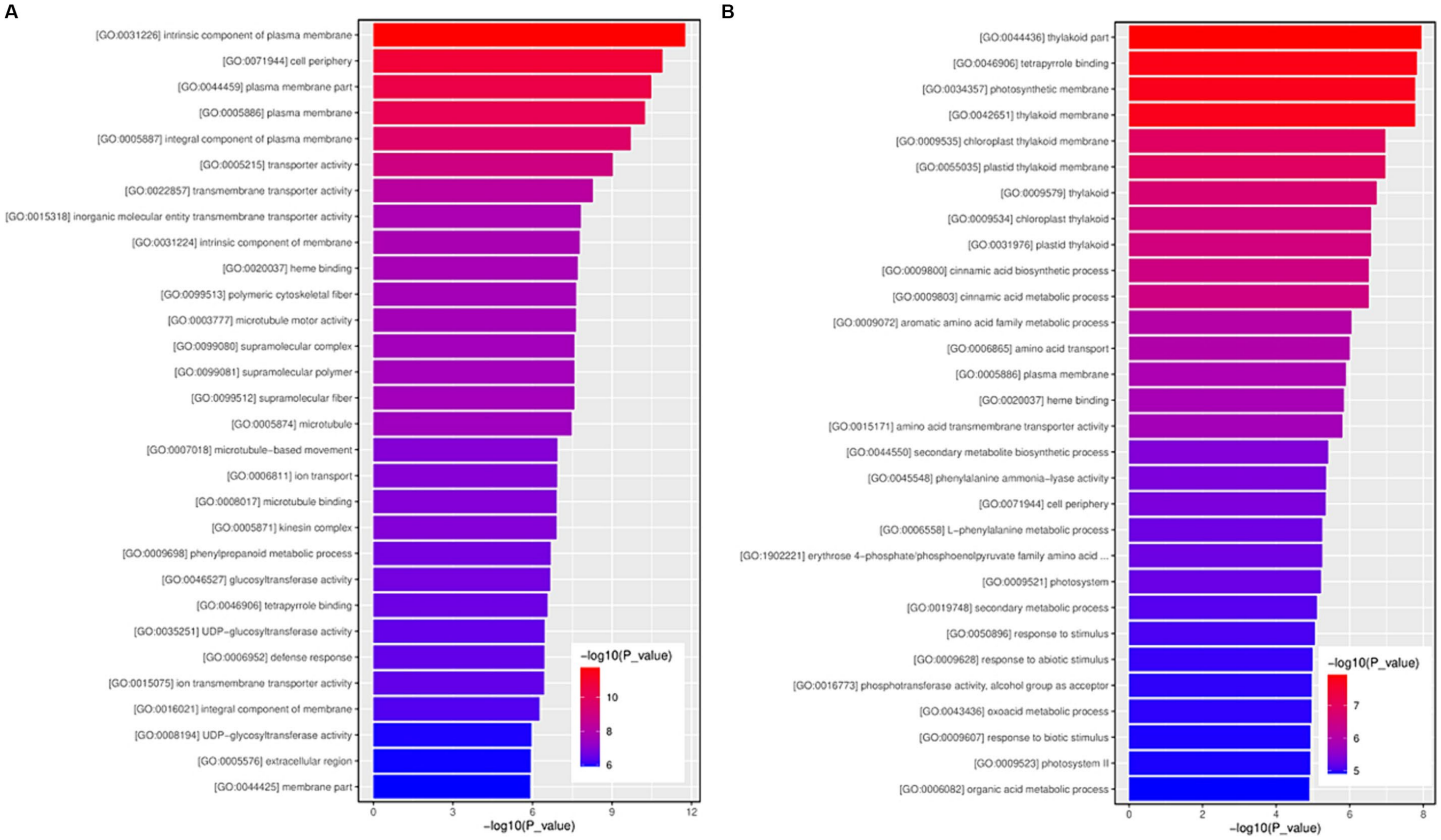
The GO function enrichment histogram of significantly differentially expressed genes. **(A)** The GO function enrichment histogram of up-regulated genes. **(B)** The GO function enrichment histogram of down-regulated genes. Each column in the figure is a GO term, and the horizontal coordinate text represents the name of the GO. The height of the column, also known as the abscissa, represents the significance of enrichment, known as *p*-value. The darker the color, the more significant the enrichment of the function. The color gradient on the right represents the size of *p*-value.

GO enrichment analysis of 3,265 upregulated and 1,916 downregulated genes was performed to study the general functions of the DEGs. The GO enrichment histogram of DEGs intuitively reflects DEG distribution in GO terms enriched in biological processes, molecular functions, and cellular components (Figure 6). Regarding biological process, the most significantly enriched GO terms for the upregulated DEGs were GO:0007018 (microtubule-based movement), followed by GO:0006811 (ion transport) and GO:0009698 (phenylpropanoid metabolic process), etc. (Figure 6A and Supplementary Table S5). The most significantly enriched GO terms for the downregulated DEGs were GO:0009800 (cinnamic acid biosynthetic process), followed by GO:0009803 (cinnamic acid metabolic process) and GO:0009072 (aromatic amino acid family metabolic process), etc. (Figure 6B and Supplementary Table S6).

Concerning molecular function, the most significantly enriched GO terms for the upregulated DEGs were GO:0005215 (transporter activity), followed by GO:0022857 (transmembrane transporter activity) and GO:0015318 (inorganic molecular entity transmembrane transporter activity), etc. (Figure 6A and Supplementary Table S5). The most significantly enriched GO terms for the downregulated DEGs were GO:0046906 (tetrapyrrole binding), followed by GO:0020037 (heme binding) and GO:0015171 (amino acid transmembrane transporter activity), etc. (Figure 6B and Supplementary Table S6).

For cellular components, the most significantly enriched GO terms for the upregulated DEGs were GO:0031226 (intrinsic component of plasma membrane), followed by GO:0071944 (cell periphery) and GO:0044459 (plasma membrane part), etc. (Supplementary Table S4) (Figure 6A and Supplementary Table S5). The most significantly enriched GO terms for the downregulated DEGs were GO:0044436 (thylakoid part), followed by GO:0034357 (photosynthetic membrane) and GO:0042651 (thylakoid membrane), etc. (Figure 6B and Supplementary Table S6).

### 3.7 KEGG pathway enrichment analysis of DEGs

**Figure 7:**
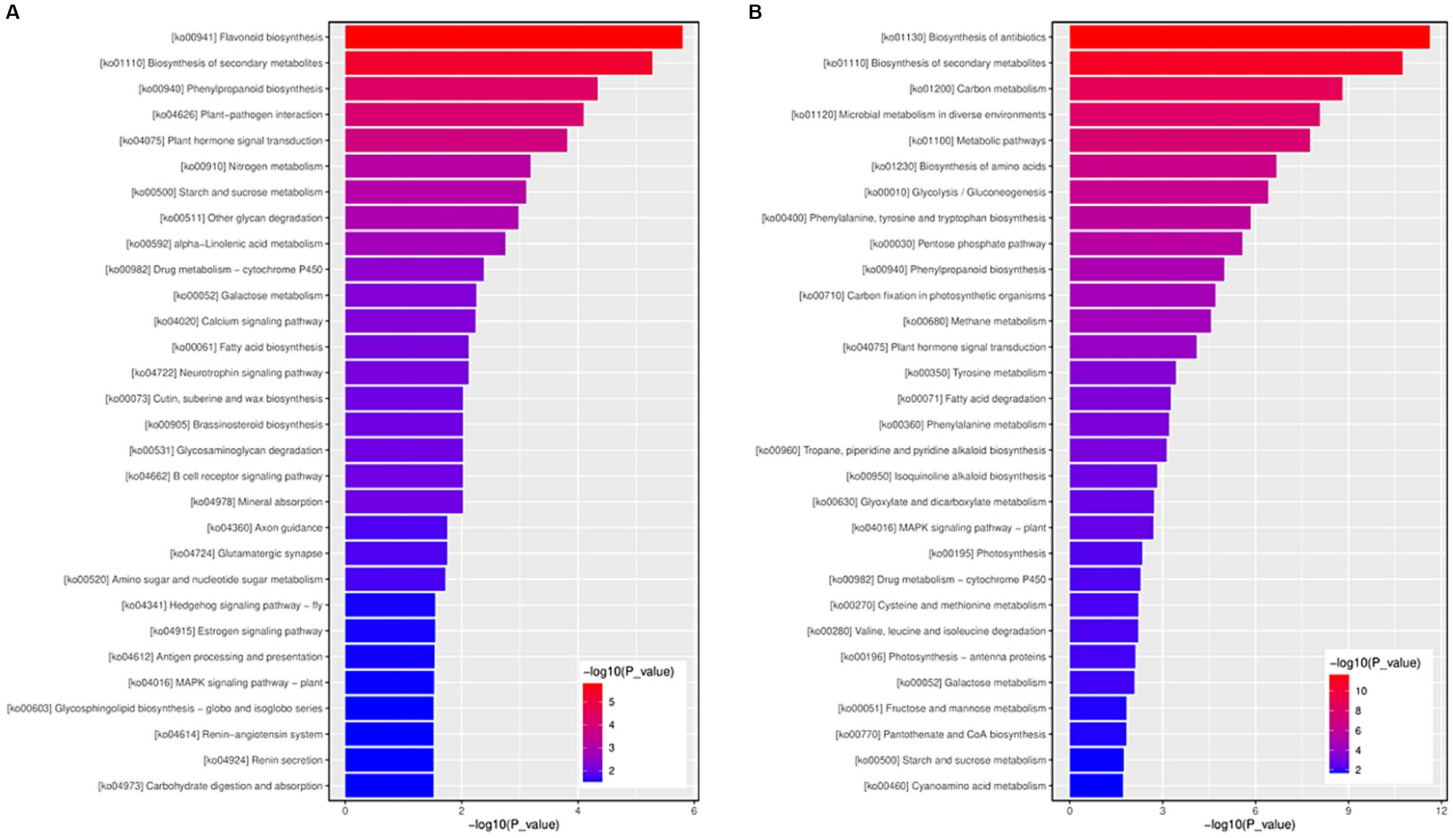
The KEGG function enrichment histogram of significantly differentially expressed genes. **(A)** The KEGG function enrichment histogram of up-regulated genes. **(B)** The KEGG function enrichment histogram of down-regulated genes. Each column in the figure represents a KEGG path, and the horizontal axis text represents the name of the KEGG. The height of the column, the abscissa, represents the significance of enrichment (*p*-value). The darker the color, the more significant the enrichment of the KEGG path. The right color gradient represents the size of *p*-value.

Adjusted *p*-values <0.05 were set as the threshold to determine the significant KEGG pathways of DEGs. Nine and 22 KEGG pathways of upregulated and downregulated DEGs, respectively, were significantly enriched (Supplementary Tables S7, S8). For the upregulated DEGs, the most significantly enriched DEGs KEGG pathways were associated with ko00941 (flavonoid biosynthesis), ko01110 (biosynthesis of secondary metabolites), and ko04075 (plant hormone signal transduction). (Figure 7A and Supplementary Table S7). Among the downregulated DEGs, the most significantly enriched DEGs KEGG pathways were ko01130 (biosynthesis of antibiotics), ko01110 (biosynthesis of secondary metabolites), and ko01200 (carbon metabolism) (Figure 7B and Supplementary Table S8). Among the 22 significantly enriched KEGG pathways, 20 were associated with metabolism and the remaining pathways were related to environmental information processing.

### 3.8 qRT-PCR of DEGs

**Figure 8:**
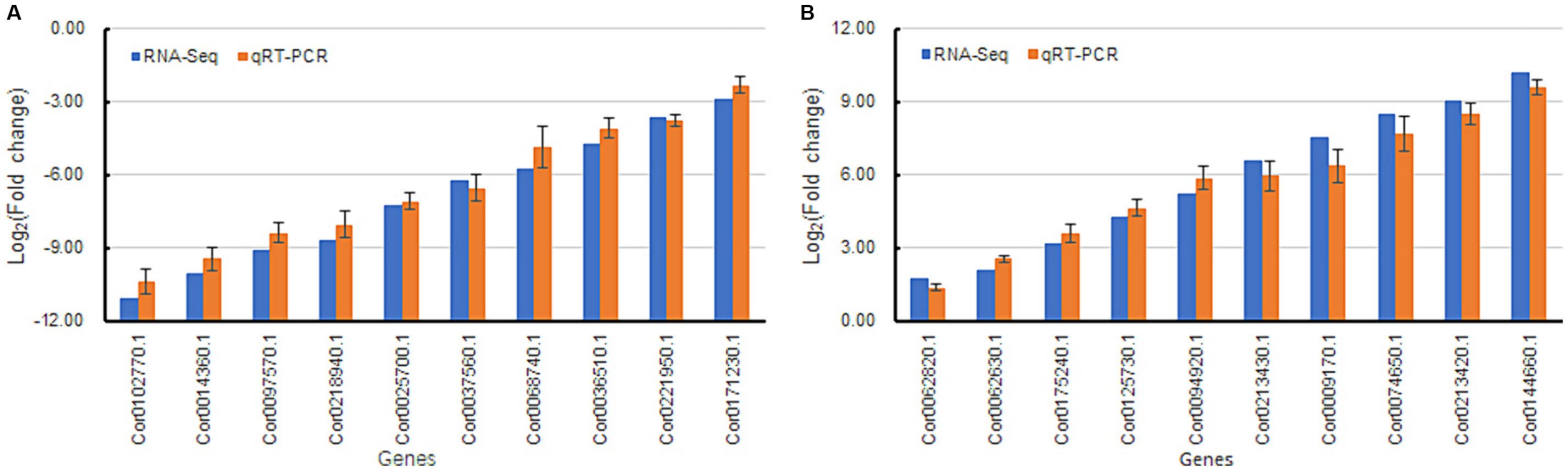
Validation of differentially expressed genes by qRT-PCR analysis. *Beta actin* was used as a reference gene in the analysis. Fold change refers to the value obtained by dividing the expression level of sample My by the expression level of sample Ck. Bars represent mean ± standard deviation (*n* = 3). RNA-seq and qRT-PCR indicated that the gene fold change obtained was acquired by transcriptome sequencing and real-time quantitative PCR, respectively. **(A)** indicates downregulated genes, and **(B)** represents upregulated genes.

Twenty DEGs (10 each of upregulated and downregulated) were randomly chosen for qRT-PCR analysis. Among the 20 DEGs, the fold changes (FCs) of those obtained from RNA-seq and qRT-PCR were well correlated (Figures 8A,B), suggesting that the expression patterns from the qRT-PCR analysis were consistent with those obtained from RNA sequencing. Thus, the reliability of the RNA-seq data was validated.

### 3.9 Immunohistochemical analysis of auxin and ARF9

**Figure 9:**
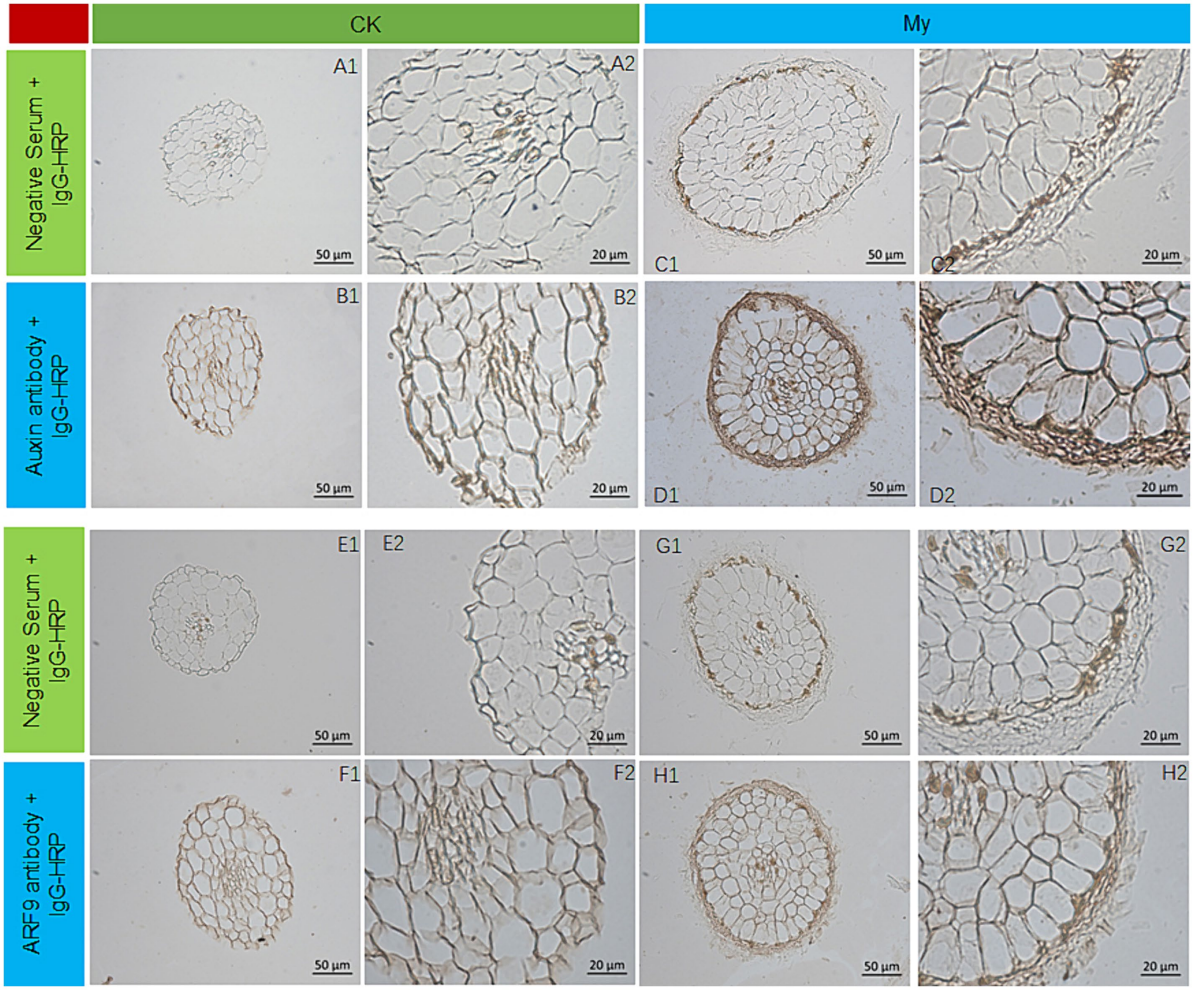
Auxin and ARF9 expression by immunohistochemical analysis. **(A1)** CK, negative control. **(A2)** Enlarged view of A1. **(B1)** CK, the first antibody is auxin antibody. **(B2)** Enlarged view of B1. **(C1)** My, negative control. **(C2)** Enlarged view of C1. **(D1)** My, the first antibody is auxin antibody. **(D2)** Enlarged view of D1. **(E1)** CK, negative control. **(E2)** Enlarged view of E1. **(F1)** CK, the first antibody is ARF9 antibody. **(F2)** Enlarged view of F1. **(G1)** My, negative control. **(G2)** Enlarged view of G1. **(H1)** My, the first antibody is ARF9 antibody. **(H2)** Enlarged view of H1. CK, non-inoculated control. My, treatments inoculated with *S. bovista* and formed mycorrhiza.

Differential transcriptome analysis of Ck and My revealed many DEGs related to auxin synthesis and signal transduction, suggesting that the regulation of auxin ectomycorrhizal fungi *S. bovista* on root development may be related to auxin. To support this, immunohistochemical analyses of Ck and My treatments were conducted using antibodies against auxin and ARF9. We conducted immunohistochemical analysis of auxin *in vitro* cultured *S. bovista*. When there was no auxin in the culture medium, the growth of *S. bovista* was very slow, and the presence of auxin could not be observed in the hypha (Supplementary Figures S1A, B1,B2); when auxin was present in the culture medium, the growth rate of *S. bovista* was significantly promoted, and high concentrations of auxin could be observed in the hypha (Supplementary Figures S1A, C1,C2). These results indicated that the *S. bovista* cannot synthesize auxin itself. Auxins in the root tips of non-inoculated plants were primarily distributed on the cell walls (Figures 9A1,A2, B1,B2). Compared with the non-mycorrhizal root tips, the distribution and expression levels of auxin in the mycorrhizal roots were significantly different. *S. bovista* formed a mantle at the root tip of the hazel, and auxin was primarily distributed in the mantle, followed by the cell wall, with the lowest concentration within the cell (Figures 9C1,C2, D1,D2). Immunohistochemical analysis of ARF9 showed that in the non-mycorrhizal control, ARF9 was expressed in the cell walls of the epidermis and cortex (Figures 9E1,E2, F1,F2). The expression level of ARF9 in the mycorrhizae was significantly higher than that in the non-mycorrhizal control. Compared with the epidermal and cortical cells of mycorrhiza, ARF9 was primarily distributed in the mantle, followed by the cell wall (Figures 9G1,G2, H1,H2). After the formation of mycorrhiza at hazelnut root tips, the expression levels of auxin and ARF9 significantly increased in *S. bovista*. In the My treatment, auxin and ARF9 secreted by the root tips actively entered the outside mantle.

## 4 Discussion

### 4.1 *Scleroderma bovista* colonizes hazelnut plants’ roots and thus boosts their growth behavior

Most ectomycorrhizal fungi are macro fungi. *S. bovista* is an edible medicinal fungus with hemostatic and detoxifying effects and a high medicinal value. Its tender fruiting body is a delicious mushroom. To form edible fruiting bodies, *S. bovista* needs to form a symbiotic relationship with host trees (Zhang et al., 2021). Therefore, the symbiotic relationship established between the *S. bovista* and the hazelnut tree benefits the growth and development of the hazelnut tree, benefits the development of the under-forest economy in the hazelnut orchard, and increases cultivation benefits. Previously, we discovered naturally formed ectomycorrhizal fungi in the hazelnut production area of Northeast China. Outdoor-potted hazelnut seedlings were inoculated with *S. bovista*, which promoted the growth of their aboveground and underground parts (Cheng et al., 2023). It is believed that *S. bovista* and hazelnut trees in the hazelnut production area of Northeast China have similar habitats (Cheng et al., 2023). *S. bovista* can form a stable symbiotic relationship with the hazelnut root system in the production area. It has been speculated that it may be crucial in promoting root development and plant growth and improving plant stress resistance. We inoculated potted hazelnut seedlings with *S. bovista*, and confirmed that the inoculation resulted in a significant increase in root biomass compared to non-inoculated controls. The length, projection area, surface area, volume, forks, and diameter of the inoculated seedlings root were 1.13–2.48 times higher than those of the control, suggesting a more developed root system in *S. bovista* inoculated treatments, consistent with our previous study (Cheng et al., 2023). Transcriptomic analysis of the non-mycorrhiza control and mycorrhiza treatment was compared in this study to elucidate the possible underlying mechanism by which *S. bovista* promotes the growth and development of hazelnut seedlings and to provide a scientific basis for developing novel cultivation techniques and ectomycorrhizal agents for hazelnuts.

### 4.2 Candidate DEGs that may be involved in transportation in hazel ectomycorrhiza

Transcriptome sequencing and DEGs identification revealed that the FPKM values of genes in the My treatments were higher than those in the control. Among the 5,181 DEGs, the number of upregulated DEGs was 1.7 times higher than that of the downregulated DEGs. These results indicate that symbiosis between *S. bovista* and hazelnut roots was more conducive to the promotion of gene expression. The GO enrichment analysis for the upregulated genes indicated that the most significantly enriched GO terms for the regulated DEGs included GO: 007018 (microtubule-based movement) and GO: 006811 (ion transport), suggesting that the symbiosis between *S. bovista* and hazelnut roots benefits cell division and ion transport. The most significantly enriched GO terms for the downregulated DEGs were GO: 0044436 (thylakoid part) and GO:0034357 (photosynthetic membrane), suggesting that the symbiosis between *S. bovista* and hazelnut roots caused the downregulation of genes related to photosynthesis. The root system grows in the dark soil and cannot undergo photosynthesis. The downregulation of photosynthesis-related genes in the root system benefits plants to save energy and promote root growth.

ATP-binding cassette (ABC) proteins contain ATP-binding domains (NBD/NBF). The ABC protein family is large, and the ABC proteins carrying transmembrane domain are called ABC transporter proteins (Theodoulou, 2000; Uroz and Oger, 2017). There are eight plant *ABCA*, *ABCG*, and *ABCI* subtypes, which are primarily involved in the active transport of heavy metals, antibiotics, and small-molecule hormones (Mentewab and Stewart, 2005; Kretzschmar et al., 2011; Borghi et al., 2015; Hwang et al., 2016; Ofori et al., 2018). In *Arabidopsis*, *ABCG31* is involved in regulating sterol accumulation on pollen surfaces (Geisler et al., 2017). Brassinosteroids are a class of plant-specific steroid hormones, and they are reportedly crucial in plant root development (Choi et al., 2014). Significantly, two DEGs encoding *ABCG31* were upregulated 9.59 and 7.25 folds in My treatment, and their orthologs in other plants were critical in sterol and small molecule hormone transport (Table 1). This suggests that *ABCG31* may affect the root development in My treatment by regulating the steroid (Brassino) transport.

**Table 1:** Genes of interest that may be involved in transportation in hazel ectomycorrhiza.

Gene ID	Expression_My	Expression_Ck	Log_2_FC	*p*_adj_	Subcluster	Annotation
Cor0123210.1	7.72	0	9.59	9.33 × 10^−17^	2	*ABCG31*
Cor0200160.1	5.00	0	8.97	8.59 × 10^−22^	2	*ACA13*
Cor0075230.1	4.74	0	8.89	5.54 × 10^−13^	2	*NPF4.6*
Cor0136210.1	4.43	0	8.79	7.31 × 10^−15^	2	*ZIP1*
Cor0102210.1	4.29	0	8.75	4.37 × 10^−15^	2	*PHT1;9*
Cor0078070.1	15.15	0.03	8.72	5.31 × 10^−17^	1	*At3g43660*
Cor0141460.1	3.83	0	8.59	2.16 × 10^−15^	2	*NRT2.7*
Cor0168500.1	2.86	0	8.16	4.75 × 10^−15^	2	*NPF5.5*
Cor0122820.1	2.63	0	8.05	4.62 × 10^−13^	2	*GONST2*
Cor0093590.1	15.76	0.07	7.55	1.29 × 10^−64^	1	*NPF5.9*
Cor0066370.1	180.87	1.01	7.46	0	6	*AMT1;2*
Cor0195570.1	1.61	0	7.34	6.36 × 10^−10^	2	*ACA13*
Cor0123220.1	4.37	0.02	7.25	3.84 × 10^−12^	2	*ABCG31*
Cor0147760.1	16.03	0.15	6.66	7.01 × 10^−83^	1	*AAP3*
Cor0000060.1	97.40	1.04	6.54	5.60 × 10^−107^	6	*COPT1*
Cor0082250.1	7.84	0.29	4.71	5.34 × 10^−9^	2	*SWEET4*
Cor0133540.1	32.74	3.65	3.16	5.34 × 10^−271^		*BOR2*
Cor0117360.1	15.46	4.67	1.72	2.35 × 10^−77^	2	*AKT1*

*AAP3, amino acid permease 3; ABCG, ABC transporter G family member; ACA, calcium-transporting ATPase; AKT1, potassium channel AKT1; AMT1;2, ammonium transporter 1 member 2; At3g43660, vacuolar iron transporter homolog 4; BOR2, boron transporter2; COPT1, copper transporter 1; GONST2, GDP-mannose transporter GONST2; NPF4.6, PTR FAMILY 4.6; NPF5.5, NRT1/PTR FAMILY 5.5; NPF5.9, NRT1/PTR FAMILY 5.9; NRT2.7, high affinity nitrate transporter 2.7; PHT, inorganic phosphate transporter; SWEET4, bidirectional sugar transporter SWEET4; ZIP1, zinc transporter 1.*

In *Arabidopsis*, *calcium-transporting ATPase 13* (*ACA13*) is involved in vegetative growth and immune processes, and its mutation causes seedling death upon bolting, suggesting an essential role in plant growth (Nolan et al., 2023). Two DEGs encoding *ACA13* were upregulated 8.97 and 7.34 folds in the My treatment, and their orthologs in other plants were crucial regulators in calcium transport and plant growth (Table 1). This suggests that *ACA13* may affect root development during My treatment by regulating calcium transport, vegetative growth, and immune processes.

Nitrate is the primary form of nitrogen absorbed by most terrestrial plants. It is a nutrient that makes up biomolecules and a signaling molecule that regulates plant growth and developmental processes. NPF5.5, NPF4.6, and NRT2.7 are nitrate transporters (Mentewab and Stewart, 2005; Borghi et al., 2015). NPF4.6 transports abscisic acid and nitrate (David et al., 2014; Léran et al., 2015; Yu et al., 2018). Numerous studies have shown that abscisic acid (ABA) promotes root growth, increases stress resistance under moderate stress, and is essential for maintaining root growth under normal conditions (Kanno et al., 2013). Three DEGs encoding *NPF5.5*, *NPF4.6*, and *NRT2.7* were highly upregulated in the My treatment, and their orthologs in other plants had critical regulatory roles in nitrate and ABA transport (Table 1), suggesting that they may affect root development in the My treatment through this transport regulation mechanism.

Similarly, *ZIP1* participates in zinc transport, accumulation, and partitioning (Teng et al., 2023). *PHT1;9* is involved in inorganic phosphate acquisition and transport in the root system (Gaitán-Solís et al., 2015). *At3g43660* and *NPF5.9* are involved in iron transport (Gollhofer et al., 2011; Remy et al., 2012). *COPT1* is involved in copper transport, and its overexpression slightly increases the endogenous iron concentration in rice grains (Chen et al., 2021). *GONST2* is a nucleotide sugar transporter that provides GDP-mannose for glycosyl inositol phosphoryl ceramide glycosylation (Andrés-Bordería et al., 2017). *AMT1;2* is involved in ammonium uptake by *Arabidopsis* roots (Jing et al., 2021). *AAP3* is essential in amino acid transport (Giehl et al., 2017). In soybean, *GsBOR2* is crucial in the efflux of boron from cells, and its overexpression in *Arabidopsis* generates longer roots than those in wild-type plants (Marella et al., 2013). *SWEET4* is a bidirectional sugar transporter (Duan et al., 2018; Andargie and Li, 2019). AKT1 is a potassium channel protein, and the overexpression of *GmAKT1* in *Arabidopsis* promotes root length and K^+^ concentration (Liu et al., 2016). DEGs encoding *ZIP1*, *PHT1;9*, *At3g43660*, *COPT1*, *GONST2*, *AMT1;2*, and *SWEET4* were highly upregulated in the My treatment, and their orthologs in other plants had critical regulatory roles in zinc, phosphate, nucleotide sugar, ammonium, amino acid, and sugar transport (Table 1), suggesting that they may significantly affect plant growth and root development in the My treatment by regulating elements, ABA, and sugar transport processes.

### 4.3 Candidate DEGs that may be involved in root development and branching in ectomycorrhiza of hazel

Auxins are vital phytohormones with essential effects on plant growth, development, and rooting. The *Arabidopsis WALLS ARE THIN1* (*WAT1*) gene family comprises 46 members, and *WAT1* is believed to be involved in auxin signaling and secondary cell wall formation in stem fibers. *wat1-1* mutation causes a significant downregulation of auxin-related gene expression, decreasing auxin content in the stem (Feng et al., 2012). WAT1 is an auxin transporter protein on the vacuole membrane, which is crucial in regulating plant vacuolar auxin homeostasis (Ranocha et al., 2010). Significantly, 14 DEGs encoding WAT1 were >1 fold upregulated in My treatment, and their orthologs in other plants are critical in auxin homeostasis, auxin signaling, and secondary cell wall formation. The three most highly regulated DEGs are listed in Table 2. This suggests that *WAT1* may affect root development during My treatment by regulating auxin homeostasis and signaling.

**Table 2:** Genes of interest that may be involved in root development and branching in ectomycorrhiza of hazel.

Gene ID	Expression_My	Expression_Ck	Log_2_FC	*p*_adj_	Subcluster	Annotation
Cor0070290.1	45.37	0	12.15	8.67 × 10^−28^	6	*WAT1*
Cor0001400.1	11.76	0	10.20	3.91 × 10^−20^	1	*WAT1*
Cor0022150.1	25.75	0.21	6.90	9.15 × 10^−29^	1	*WAT1*
Cor0069300.1	1.78	0.00	7.48	9.77 × 10^−12^	2	*YUC5*
Cor0116580.1	5.66	0.58	3.27	4.89 × 10^−36^	2	*YUC2*
Cor0113600.1	1.28	0.28	2.16	3.53 × 10^−5^	2	*YUC6*
Cor0100160.1	5.55	0.16	5.02	3.52 × 10^−44^	2	*AUX1*
Cor0020280.1	6.36	2.92	1.12	2.44 × 10^−10^	2	*AUX1*
Cor0178640.1	24.18	7.49	1.69	2.26 × 10^−82^	2	*TIR1*
Cor0086990.1	30.60	0.78	5.28	7.09 × 10^−65^	1	*AUX/IAA*
Cor0140460.1	18.49	1.16	3.99	3.14 × 10^−63^	2	*AUX/IAA*
Cor0096370.1	77.47	5.20	3.90	1.11 × 10^−192^	1	*AUX/IAA*
Cor0110070.1	178.22	47.05	1.92	4.57 × 10^−266^	2	*AUX/IAA*
Cor0194170.1	195.23	37.11	2.40	6.10 × 10^−172^	2	*AUX/IAA*
Cor0140470.1	248.46	71.24	1.80	1.08 × 10^−175^	2	*AUX/IAA*
Cor0156840.1	170.25	24.56	2.79	0	2	*ARF9*
Cor0082950.1	1.05	0.04	4.28	8.92 × 10^−11^	2	*GH3.6*
Cor0060400.1	251.23	13.61	4.21	0	1	*GH3.1*
Cor0038980.1	5.62	0.70	3.00	2.70 × 10^−14^	2	*SAUR*
Cor0159730.1	5.48	2.30	1.25	0.00	2	*IPT3*
Cor0208640.1	1.90	11.50	−2.59	0.00	3	*PUP3*
Cor0178800.1	121.73	19.58	2.64	0.00	2	*ENT3*
Cor0123210.1	7.72	0.00	9.59	0.00	2	*ABCG31*
Cor0123220.1	4.37	0.02	7.25	0.00	2	*ABCG31*
Cor0023350.1	2.06	0.07	4.76	0.00	2	*ABCG22*
Cor0017160.1	10.18	1.14	3.15	0.00	2	*ABCG24*
Cor0002720.1	31.81	75.04	−1.24	2.68 × 10^−134^	3	*EIN2*
Cor0027360.1	4.27	0.00	8.74	1.14 × 10^−12^	2	*ROPGEF11*
Cor0060040.1	364.79	90.43	2.01	1.57 × 10^−277^	2	*HKL*

*ABCG, ABC transporter G family member; ARF, auxin response factor; AUX1, auxin influx carrier; EIN2, ethylene-insensitive protein 2; ENT3, equilibrative nucleotide transporter 3; HKL, hexokinase like; GH3, Gretchen Hagen3; IAA, auxin-responsive protein IAA; IPT3, adenylate isopentenyltransferase 3; PUP3, purine permease 3; ROPGEF, Rop guanine nucleotide exchange factor; SAUR, small auxin-up RNA; TIR1, transport inhibitor response 1; WAT1, WALLS ARE THIN1; YUC, indole-3-pyruvate monooxygenase YUCCA*.

Auxin synthesis involves tryptophan-and non-tryptophan-dependent pathways. Plants primarily complete the synthesis of tryptophan to auxin through the tryptamine, indole-3-pyruvate (IPA), and indole-3-acetonitrile pathways, depending on the intermediate products. In the IPA pathway, the rate-limiting step for converting IPA to indoleacetic acid (IAA) is categorized by YUCCA (Kim et al., 2013; Ranocha et al., 2013; Chen et al., 2014). Simultaneous loss of mutations in *YUC5*, *YUC3*, *YUC7*, *YUC8*, and *YUC9* caused short and agravitropic primary roots, implying vital regulatory roles for *YUC5* in root development (Kim et al., 2013). Auxin regulates stem growth rate, inhibits lateral buds, and promotes rooting. Three DEGs encoding *YUC5*, *YUC2*, and *YUC6* were highly upregulated in the My treatment. We have completed genome sequencing of *S. bovista*, and its genome sequence was uploaded to the public database NCBI with the accession number of PRJNA1048296. We did not find any genes related to auxin synthesis in its genome. Consistent with these, after *S. bovista* were cultured on auxin free medium, no auxin could not be detected in the hypha of *S. bovista using* immunohistochemical analysis. After symbiosis with hazelnut roots, immunohistochemical analysis showed that auxin expression levels in the mycorrhiza complex increased significantly, further supporting that *S. bovista* promotes auxin biosynthesis in hazelnut roots. Thus, the orthologs of *YUC5*, *YUC2*, and *YUC6* in other plants significantly regulate auxin biosynthesis and root development (Table 2), suggesting that they may promote root development during My treatment by regulating auxin biosynthesis.

In *Arabidopsis*, AUX1 (auxin influx carrier) functions as a permease-like regulator of root gravitropism (Bennett et al., 1996; Li et al., 2015). The Aux/IAA-TIR1-ARF complex is crucial in activating auxin signal transduction. TIR1 is in the nucleus and acts as an auxin receptor, binding to auxin molecules to initiate auxin signaling (Vandenbussche et al., 2010). When auxin is absent or at a low concentration, AUX/IAA forms heterodimers with ARF, preventing ARF from binding to the promoters of downstream auxin-induced genes and inhibiting gene transcription (Tian et al., 2002; Hayashi et al., 2008). However, when plant cells are stimulated by external or internal auxins, IAA binds to TIR1 to form a TIR1-IAA complex. IAA binding stabilizes the interaction between the second domain of TIR1 and Aux/IAA proteins, causing their degradation (Tian et al., 2002; Hayashi et al., 2008). This releases the ARF protein and promotes the transcription of downstream genes, activating the auxin response. The GH3 (Gretchen Hagen3) bind auxin and amino acids, controlling plant auxin levels (Hagen and Guilfoyle, 2002). The 20 *GH3* genes in *Arabidopsis* can be divided into three subfamilies, with *GH3.1* and *GH3.6* belonging to Subfamily II. Changes in free auxin content caused by the expression of *AtGH3s* affect plant architecture and root development in *Arabidopsis*. *AtGH3.6* overexpression caused *Arabidopsis dfl1-D* dwarf lines with shorter stems and fewer lateral roots than the wild type (Wang et al., 2023). Auxin regulates the expression of many genes, including three types of early auxin-responsive gene families: *Aux/IAA*, *GH3*, and *small auxin-up RNA* (*SAUR*) (Nakazawa et al., 2008). Among these gene families, *SAURs* are the most rapidly responsive to auxin. SAURs are primarily involved in regulating auxin synthesis and transportation (Liscum and Reed, 2002). DEGs encoding *AUX1*, *Aux/IAA*, *TIR1*, *ARF*, *GH3*, and *SAURs* were differentially expressed in My treatment, with most genes being upregulated. Consistent with this, immunohistochemical analysis of *ARF9* showed that its expression in the mycorrhiza complex was promoted by symbiosis between *S. bovista* and hazel roots. The orthologs of *AUX1*, *Aux/IAA*, *TIR1*, *ARF*, *GH3*, and *SAURs* in other plants regulate auxin influx and its signal transduction, level, synthesis, and transportation (Table 2), suggesting that they may significantly affected root development in the My treatment by regulating auxin synthesis, transportation, and signal transduction processes. These data suggest that auxin biosynthesis and signal transduction pathways are activated in symbiosis with *S. bovista*, benefiting hazel root development.

We found that ARF9 had the highest expression level in the mantle. *ARF* was not in the genome of *S. bovista*. All hazelnut *ARF* sequences were obtained from the hazelnut genome database using SignalP software (v5.0)^6^ (Wang et al., 2020). Signal peptide prediction was conducted, and potential signal peptide cleavage sites were found in the coding sequence of *ARF* (*Cor0105760.1*). The sequence of ARF9 is homologous to it. It is speculated that after the expression of Cor0105760.1, the protein in the hazelnut root system is guided by the signal peptide into the extracellular space and enters the symbiotic mantle to regulate the symbiotic process between *S. bovista* and hazelnut. This discovery may mean that plant ARF proteins enter the microbial hyphae through a mechanism that regulates symbiosis between microorganisms and plant roots, providing a novel idea for analyzing the symbiosis mechanism between ectomycorrhizal fungi and plant hosts.

Cytokinins are primarily synthesized at the root tip and transported to other parts of the root and aboveground parts. The key rate-limiting step in cytokinin synthesis is catalyzed by *IPT* (Petersen et al., 2011). Purine permeaminase (PUP) and equilibrative nucleotide transporter 3 (ENT3) are transporters that can actively transport cytokinin (Galichet et al., 2008). The three primary types of cytokinin transporter proteins belong to the *PUP*, *ENT*, and *ABCG* families and are membrane-localized (Wang et al., 2023). DEGs encoding *IPT3*, *PUP3*, *ENT3*, *ABCG31*, *ABCG22*, and *ABCG24* were differentially expressed in the My treatment, and most were upregulated. The orthologs of *IPT3*, *PUP3*, *ENT3*, *ABCG31*, *ABCG22*, and *ABCG24* in other plants are essential regulators of cytokinin biosynthesis and transport, implying that they markedly affected the root branch in the My treatment by regulating cytokinin biosynthesis and transport processes.

After symbiosis formation between exogenous fungi and their hosts, the root structure of plants undergoes significant changes, including the formation of multiple thick and short lateral roots to increase the surface area for ectomycorrhizal fungi colonization. The root hairs of plant roots decrease because their function is replaced by the fungal sheath formed by ectomycorrhizal fungi (Romanov and Schmülling, 2021). The complex PIRF1, formed by a combination of Phytochrome and ROP guanine nucleotide exchange factor (RopGEF1), regulates root development by activating the Rho-like GTPases of plants in the cytoplasm. Mutations in PIRF1 cause delayed root elongation and irregular root hair formation, suggesting that RopGEF1 negatively regulates primary root development (Tikhonovich and Provorov, 2007). Transgenic lines that overexpress hexokinase-like1 (HKL1) suggest that hexokinase is a negative regulator of root hair development (Shin et al., 2010). ein2 mutants cause shorter root hairs phenotype (Karve and Moore, 2009). After symbiosis formation between *S. bovista* and hazel root, the function of root hairs is replaced by a fungal sheath, and mycorrhizal roots are characterized by more short roots, fewer long roots, and fewer root hairs. The orthologs of ROPGEF1 and HKL1 in other plants are vital regulators in the development of prime root and root hair (Table 2); as negative regulators, its upregulation may inhibit the development of long root formation and root hair of My treatment; ein2 is a positive regulator in root formation and root hair, it was upregulated in the My treatment. Expression changes in these sets of DEGs caused fewer root-hair phenotypes.

In the symbiotic establishment stage, hyphae physically contact the surface of the plant roots, wrap around the root tip, form hyphal sheaths, and invade the plant root cortex cells to form a Hartig network. During this process, the symbiotic fungi avoid host immune rejection. Recent studies have shown that the genetic engineering of individual plant host genes can selectively inhibit or allow the colonization of specific fungi (Yu et al., 2015; Qiao et al., 2021; Wang et al., 2022). It has been determined that the *G-type lectin receptor-like kinase* (*PtLecRLK1*) in *Populus tomentosa* determines the colonization of *Laccaria bicolor* in the roots. When *PtLecRLK1* was transferred to the non-host plants *Arabidopsis* and switchgrass, the plant allowed *Laccaria bicolor* to invade the root system and form a Hartig network, establishing a symbiotic relationship (Labbé et al., 2019; Qiao et al., 2021). This result suggests that PtLecRLK1 is a positive regulatory factor of the symbiosis between *Laccaria bicolor* and plants. DEGs encoding *G-type lectin S-receptor-like serine/threonine-protein kinase* were differentially expressed in My treatment, including 11 upregulated and 28 downregulated DEGs (Supplementary Table S3). Among these, *Cor0108530.1*, *Cor0099180.1*, and *Cor0133230.1* were highly upregulated. The orthologs of G-type lectin S-receptor-like serine/threonine-protein kinases in other plants are crucial in regulating the symbiosis between ectomycorrhizal fungi and plants, suggesting their potential significance in the ectomycorrhiza development of My treatment.

## 5 Conclusion

After inoculation with *S. bovista*, the hazelnut root system formed an ectomycorrhizal symbiosis with the fungus. Ectomycorrhizal fungus *S. bovista* can increase the lateral root branching of hazelnuts, increase the root absorption area, and strongly promote the growth of hazelnut seedlings. Genes related to auxin synthesis, transportation, and signal transduction have been identified, including *YUC5*, *AUX1*, *Aux/IAA*, *TIR1*, *ARF*, *GH3*, and *SAURs*. They are upregulated in mycorrhiza and may be beneficial for root growth and development. Genes related to nutrient transport has been identified, including *ZIP1*, *PHT1;9*, *At3g43660*, *COPT1*, *GONST2*, *AMT1;2*, and *SWEET4*, and they may be are involved in zinc, phosphate, nucleotide sugar, ammonium, amino acid, and sugar transport. They are upregulated in mycorrhizal fungi, and may improve plant nutrient absorption. Finally, a set of G-type lectin S-receptor-like serine/threonine-protein kinase was identified, which was upregulated in My treatment and may be involved in the symbiotic regulation of *S. bovista* and host root systems. The conclusion of this study is mainly based on transcriptome data and has not been further validated for gene mutations and overexpression, therefore the conclusion has limitations. Nevertheless, this study provides a preliminary and reasonable explanation for the promotion of hazelnut seedling growth by *S. bovista*, which provides a scientific basis for further research and development of hazelnut mycorrhizal preparations in the future.

*Scleroderma bovista* can form symbiotic ectomycorrhizal fungi with hazel roots. The mechanism through which *S. bovista* promotes hazelnut growth remains unclear. This study aimed to evaluate the effects of the ectomycorrhizal fungus *S. bovista* on the growth and development of hazelnuts and gene expression changes through comparative transcriptome analysis. After inoculation with *S. bovista*, the fungus symbiotically formed ectomycorrhiza with hazel roots. The fresh weights of the aboveground and underground parts of My treatment (inoculated with *S. bovista* and formed mycorrhiza) were much higher than those of the control, respectively. The length, project area, surface area, volume, forks, and diameter of the inoculated seedlings root were 1.13 to 2.48 times higher than those of the control. In the paired comparison, 3,265 upregulated and 1,916 downregulated genes were identified. The most significantly enriched Gene Ontology term for the upregulated differentially expressed genes was GO:0005215 (transporter activity). Immunohistochemical analysis suggested that the expression levels of auxin and auxin response factor 9 were significantly increased by *S. bovista* after the formation of mycorrhizal fungi in hazelnut root tips. These results indicate that genes related to auxin biosynthesis, transport and signaling, and transport of nutrients may contribute to root development regulation in hazel ectomycorrhiza.

## Data availability statement

The datasets presented in this study can be found in online repositories. The names of the repository/repositories and accession number(s) can be found in the article/Supplementary material.

## Author contributions

YC: Conceptualization, Writing – review & editing. SS: Writing – review & editing, Methodology. HL: Methodology, Writing – review & editing. YD: Methodology, Writing – review & editing. HH: Methodology, Writing – review & editing. QM: Methodology, Writing – review & editing. JL: Writing – review & editing, Conceptualization, Writing – original draft.

## Footnotes

^1^https://www.ncbi.nlm.nih.gov/SRA

^2^https://daehwankimlab.github.io/hisat2/

^3^http://122.9.151.76/#/map

^4^https://ccb.jhu.edu/software/stringtie/index.shtml

^5^http://mev.tm4.org/#/welcome

^6^https://services.healthtech.dtu.dk/service.php?SignalP-5.0
