# Supplementary material for: The ectomycorrhizal fungus Scleroderma bovista improves growth of hazelnut seedlings and plays a role in auxin signaling and transport
Source: Front Microbiol. 2024 Aug 7;15:1431120. doi: 10.3389/fmicb.2024.1431120 (PMC11335501; doi:10.3389/fmicb.2024.1431120)
Supplement: Supplementary file 1 [file Data_Sheet_1.PDF]

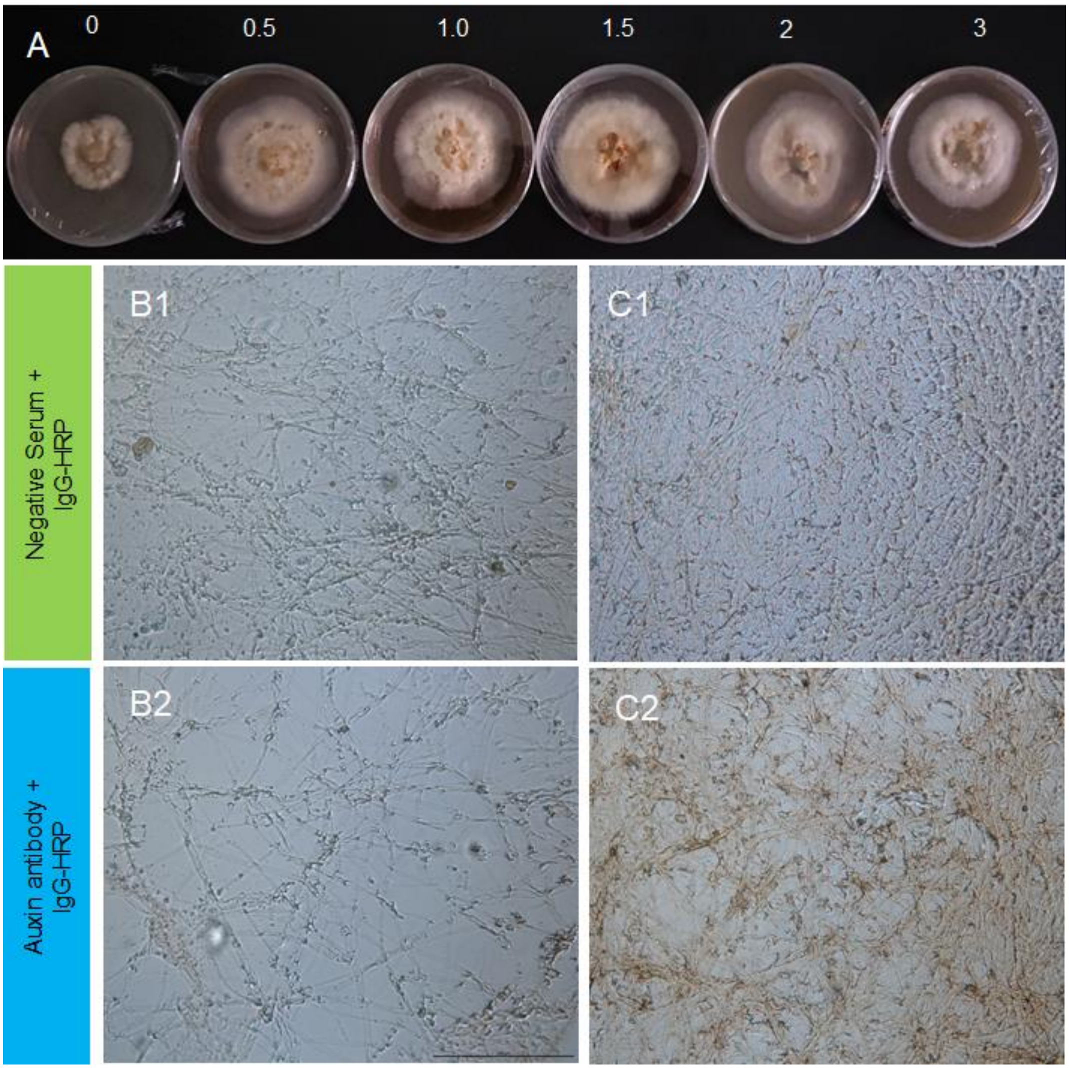

Figure S1. The effect of auxin on *Scleroderma bovista* growth and its auxin immunohistochemical localization in hyphae

A: Adding auxin to culture medium can accelerate the growth of *S. bovista* to varying degrees. The number above the culture dish represents the concentration (mg/L) of auxin in the MMN medium (Modified Melin-Norkrans Medium), and the cultivation time is 3 weeks; B1 and B2: *S. bovista* were subjected to auxin immunohistochemical localization after growing in a auxin free MMN medium, and no auxin was observed in the hypha; C1 and C2: *S. bovista* were subjected to auxin immunohistochemical localization after growing in a MMN medium containing 2.0 mg/L auxin, and high concentrations of auxin was observed in the hypha.
